# Cases of Indolent Buboes Cured by the Use of the Tobacco Ointment

**Published:** 1828-06

**Authors:** 


### INDOLENT BUBOES.

*Cases of Indolent Buboes cured by the use of the Tobacco Ointment.*
By Dr. Graham.

James Smith applied to me on the 5th of July, 1827. His complaints were phymosis, purulent discharge from the glans and prepuce, and an indurated bubo in the right groin. He stated that he had been five months ill, and that the first symptom which presented itself was gonorrhœa, and shortly afterwards the other complaints made their appearance. He also stated that he had observed no alteration in the size of the bubo for two months. I directed the antimonial solution and black wash. In the course of a few days, the discharge and swelling were entirely removed, and on retracting the prepuce there was no ulceration whatever. He stated to me that mercurial frictions, leeches, and cold lotions, had been used two months before without any good effect in dispersing the bubo. I ordered him to have a blister applied, and afterwards to have it dressed with the Ung. Hydrargyri, for the purpose of promoting the discharge and preventing its healing.

In the same manner four blisters were applied without any diminution in its size. I was then induced for the first time to try the effects of the tobacco ointment, from a hint given me of its virtues by Professor M'Clellan, of Philadelphia. I ordered him to rub in the size of a walnut of the ointment over the bubo three times a day, and, to my astonishment, in the course of ten days it was completely dispersed.

Patrick Burns applied to me on the 12th of July, 1827. His complaints were—a superficial ulcer on the prepuce, without induration; a large bubo in the right groin; and a papular eruption which extended to almost every part of his body, appearing very similar to true syphilitic blotches. He stated that he had been three months ill, and that he had undergone two courses of mercury without any good effect whatsoever on his complaints. He said that his physician had applied leeches three times to the bubo, followed by frictions with camphorated liniment, without any effect. He complained of severe pains in his shoulders and other joints. As there was no febrile action present, I directed for him the decoction of sarsaparilla, together with the antimonial solution. I also ordered him to keep lint moistened in the lotion of calomel and lime-water to the sores on the penis.

18th.—The eruption was on the decline, and his pains were considerably relieved.

22d.—The eruption had almost disappeared; there was scarcely any pains in his joints, and the sore on the penis had healed. In the course of this time the bubo evinced neither a tendency to disperse nor to suppurate. I ordered him, as in the former case, to commence rubbing in the tobacco ointment, which completely dispersed it in the course of twelve days.

William Ryan applied to me on the 20th of July, 1827. His complaints were—excoriation of the glans and prepuce, with purulent discharge; a small ulcer on the prepuce, without induration; a large bubo on the left groin; an eruption of papulæ on his face, arms, and neck; enlargement of the tonsils, with difficulty of swallowing; and pains in his shoulders and knees.

He said he had been five months diseased, and that he had been repeatedly salivated. I directed the same medicines as were employed in the preceding case with the same happy effect. The bubo was completely dispersed by the tobacco ointment in the course of two weeks.

